# Air-stable and ultrasensitive solution-cast SWIR photodetectors utilizing modified core/shell colloidal quantum dots

**DOI:** 10.1186/s40580-020-00238-3

**Published:** 2020-08-17

**Authors:** Jin-Beom Kwon, Sae-Wan Kim, Byoung-Ho Kang, Se-Hyuk Yeom, Wang-Hoon Lee, Dae-Hyuk Kwon, Jae-Sung Lee, Shin-Won Kang

**Affiliations:** 1 School of Electronics Engineering, College of IT Engineering, Kyungpook National University, 1370 Sankyuk-dong, Daegu, 702-701 Republic of Korea; 2 Advanced Semiconductor Research Center, Gumi Electronics and Information Technology Research Institute (GERI), Gumi, 39253 Republic of Korea; 3 Department of Electronic Engineering, Kyungil University, Hayang-up, 712-702 Gyeongsang buk-do Republic of Korea

**Keywords:** Short-wave infrared, Photodetectors, Quantum dot, Type-I core/shell structure, All solution-process

## Abstract

InGaAs-based photodetectors have been generally used for detection in the short-wave infrared (SWIR) region. However, the epitaxial process used to grow these materials is expensive; therefore, InGaAs-based photodetectors are limited to space exploration and military applications. Many researchers have expended considerable efforts to address the problem of SWIR photodetector development using lead sulfide (PbS) quantum dots (QDs). Along with their cost-efficient solution processability and flexible substrate compatibility, PbS QDs are highly interesting for the quantum-size-effect tunability of their bandgaps, spectral sensitivities, and wide absorption ranges. However, the performance of PbS QD-based SWIR photodetectors is limited owing to inefficient carrier transfer and low photo and thermal stabilities. In this study, a simple method is proposed to overcome these problems by incorporating CdS in PbS QD shells to provide efficient carrier transfer and enhance the long-term stability of SWIR photodetectors against oxidation. The SWIR photodetectors fabricated using thick-shell PbS/CdS QDs exhibited a high on/off (light/dark) ratio of 11.25 and a high detectivity of 4.0 × 10^12^ Jones, which represents a greater than 10 times improvement in these properties relative to those of PbS QDs. Moreover, the lifetimes of thick-shell PbS/CdS QD-based SWIR photodetectors were significantly improved owing to the self-passivation of QD surfaces.

## Introduction

Semiconductor colloidal quantum dots (QDs) are promising candidates for next-generation optoelectronic technologies [e.g., light-emitting diodes (LEDs), visible and infrared (IR) photodetectors, and photovoltaic (PV) devices] [1–5] owing to their unique properties such as their high absorption coefficients, tunable bandgaps, and multiple exciton generation effects [6–9]. The electrical and optical properties of QDs can be tuned by controlling the size of strong quantum confinement effect [10]. Among the many applications of QDs, short-wave IR (SWIR) photodetectors are widely used in bio-imaging, security, face recognition, food safety inspection, and optical communications [11–15]. The technological advancement of SWIR photodetectors is not only attractive for these diverse applications, but also for the commercialization of technologies that are safe for human eyes.

The SWIR band is in the range of 1–2.5 µm, which is widely used as a visually safe waveband. However, the only available high-efficiency photodetector (i.e., InGaAs) is expensive to produce via an epitaxial growth process; therefore, this photodetector is limited to applications in space exploration and military [16, 17]. Nevertheless, an attractive platform for low-cost photodetectors in the SWIR spectral region is provided by lead sulfide (PbS) QDs owing to their excellent photosensitivity, bandgap tunability, and solution-processability [3, 18–22]. In a study by So et al., solution-processed inorganic SWIR photodetectors were fabricated using large, highly monodispersed PbS QDs to provide good light-sensitivity, stability, and device lifetime [18]. Gurbuz et al. have examined the use of PbS colloidal QD-based photodiodes, which were formed from a photosensitive PbS layer and a Schottky contact, owing to their fast response and moderate sensitivity [23]. Hechster et al. have proposed a heterojunction structure for a PbS/TiO_2_-based SWIR photodetector, which can be operated either as a standalone detector or for SWIR absorption [24]. The SWIR photodetector revealed the existence of the PbS QDs bandgap operating in the SWIR spectral region. Meanwhile, Konstantatos et al. reported a device that employed a photoconductive mechanism [25]. In the simple fabrication method, a layer of QDs was spin-coated onto a pair of preexisting gold electrodes. The device exhibited considerable photoconductive gains (several thousand) under high (50 V) bias and, more practically, gains in the 10–100 range under few-volt biases. Although PbS QDs afford good photodetector sensitivity and fast response, they are nevertheless found to be structurally unstable at high temperatures or under severe operating conditions.

For practical application in commercial products, QDs are required to have good photo and thermal stabilities. These properties, along with device performance, have recently been improved by coating an inorganic CdS shell onto a PbS core [26–28]. Though PbS/CdS QDs have been widely applied to LEDs, they have not been widely used in solar cells and photodetectors because the charge injection efficiency decreases with an increase in shell thickness owing to the energy barrier provided by the CdS shell. Moreover, the type-I structure is not favorable for solar cell and photodetector applications with respect to charge separation and transport. Improved charge transfer properties of the core/shell structure, and increased photodetector efficiency have been achieved by replacing the long-carbon-chain ligands of QDs with a shorter ligand [29–32]. Nevertheless, because the ligand exchange process is performed several times, long processing time can induce binding to the substrate, which acts as a trap and negatively affects photodetector performance [33]. Therefore, band alignment and interfacial structures have to be engineered to balance charge transfer and surface passivation. According to Jin et al., these properties are simultaneously maximized by the “giant” core/shell/shell structure of PbS/CdS/CdS QDs [34]. The gradient interfacial layer between the PbS core and CdS shell allows excitons to partially leak into the shell, which improves the charge transfer.

To improve the charge transfer and stability of QDs, this study focuses on the synthesis of a type-I core/shell structure and its induced CdS passivation on the PbS QD surface. Improved device performance is demonstrated for an all-solution-processed air-stable SWIR photodetector. The problem of surface passivation is solved by the type-I structure in which a shell of a different wider bandgap semiconductor is grown around the core. To our knowledge, there are previous studies on such self-passivation-based SWIR photodetectors. This lack of studied may exist because the thick shell layer of the type-I structure impedes exciton dissociation, charge extraction, and transport [35]. The CdS shell generates higher photoluminescence quantum yields (PL QYs) [36] and considerably enhances photochemical and thermal stabilities [37]. The considerable effect of additional CdS shell thickness on device performance was demonstrated by comparing the current–voltage (I–V), and current density–voltage (J–V) behaviors, photoresponses, and long-term stabilities of three types of SWIR photodetectors fabricated using PbS QDs, PbS/CdS thin-shell QDs, and PbS/CdS thick-shell QDs. The device with PbS/CdS thick-shell QDs exhibited a considerable enhancement in the on/off ratio, detectivity, photoresponse, and long-term stability compared to those of a PbS QD device without a CdS shell. Particular attention was paid to the PL decay properties of PbS QDs, thin-shell PbS/CdS QDs, and thick-shell PbS/CdS QDs as a function of time to determine that PL decay spectra inevitably appear in PbS/CdS QD-based devices. By analyzing passivated QDs, we confirmed not only the photostability but also the efficient carrier dynamics and improved lifetime of SWIR photodetectors. To evaluate their performance, we fabricated solution-processable SWIR photodetectors by employing a modification of previously reported methods [38].

## Experimental details

### Materials

Lead chloride (PbCl_2_, 99.99%), sulfur (S, 99.98%), oleylamine (OLA, technical grade, 70%), cadmium oxide (CdO, 99.99%), oleic acid (OA, 90%), 1-octadecene (1-ODE, 90%), trioctylphosphine (TOP, 90%), Zinc acetate dehydrate (Zn(acet)_2_·2H_2_O, 99%), and anhydrous toluene and ethanol were purchased from Sigma-Aldrich. Potassium hydroxide (KOH, AR reagent), 2-propanol, and hexane were obtained from Duksan Pharmaceutical Co. Ltd. All chemicals were used as received, without further purification.

### Synthesis of colloidal PbS QDs

PbS QDs were prepared using similar previously reported methods with modifications [33, 38, 39]. In a typical synthesis, two separate solutions containing 1 mmol of PbCl_2_ and 0.36 mmol of sulfur in 2.4 mL and 0.24 mL of OLA, respectively, were stirred for 30 min at room temperature under Ar gas flow. Then, the PbCl_2_-OLA mixture was heated to 160 °C for 1 h before cooling to 120 °C under vacuum degassing for 20 min. Then, the prepared S-OLA solution and 225 µL of TOP were quickly injected into the reactor at elevated temperature under Ar gas flow. The reaction temperature was maintained at 100 °C for 30 min to grow PbS QDs. Synthesized QDs were purified by adding a toluene and ethanol solution, followed by centrifugation at 3,000 rpm for 10 min to separate QDs through precipitation. The supernatant liquid phase was decanted to remove the excess reagent; then, QDs were dispersed in a non-polar toluene solution at the concentration of 20 mg/mL.

### Synthesis of colloidal PbS/CdS QDs with thin shells

PbS/CdS QDs with thin shells were synthesized using the cation exchange method [39]. In a typical synthesis, 2.3 mmol of CdO in 2 mL of OA and 10 mL of 1-ODE were placed in a 100 ml three-necked flask and heated to 255 °C under high-purity Ar gas flow for 30 min. Then, the clear solution was cooled to 150 °C under vacuum for 20 min. Then, a PbS QD suspension in toluene (1 mL, absorbance = 3 at the first exciton peak) was diluted with 10 mL of toluene, bubbled with Ar for 30 min, and heated to 100 °C prior to adding a Cd/OA mixture by injection. The growth reaction was conducted at 100 °C for 30 min; then, the reaction cell was quenched with cold water. Then, ethanol was added; the suspension was centrifuged, and the supernatant was removed. Finally, PbS/CdS QDs with thin shells were dispersed in toluene at the concentration of 20 mg/mL.

### Synthesis of colloidal PbS/CdS QDs with thick shells

PbS/CdS QDs with thick shells were synthesized using a two-step cation exchange method [39, 40]. Without purifying thin-shell PbS/CdS QDs, the reaction temperature was increased to 225 °C for 2 h. The purification and dispersion of thick-shell PbS/CdS QDs was performed using the same procedure as that used for PbS QDs and thin-shell PbS/CdS QDs.

### Synthesis of ZnO nanoparticles via the sol–gel method

A modification of the sol–gel method was used for the synthesis of ZnO nanoparticles (NPs) in an alcohol solution [7, 41–45]. The solutions were prepared using dispersions of Zn(acetate)_2_·2H_2_O (2.46 g) and KOH (0.96 g) in 110 mL and 50 mL of methanol, respectively. The Zn(acetate)_2_·2H_2_O solution was placed in a 200 mL flask and heated to 60 °C, followed by the dropwise addition (1 mL/s) of a KOH dispersion. The mixture was stirred at 60 °C for 60 min, and then allowed to cool. To obtain uniform ZnO NPs, the required aging process was implemented by adding 2-propanol and hexane and allowing the mixture to stand overnight. Then, ZnO NPs were precipitated via centrifugation at 3000 rpm and re-dispersed in ethanol (30 mg/mL). To determine characteristics of the ZnO NPs, we performed a UV–visible spectrum analysis and transmission electron microscopy (TEM) evaluation (Additional file 1: Fig. S1).

### Device fabrication and characterization

The SWIR photodetectors were fabricated by spin-coating onto glass substrates which were commercially pre-coated with an indium tin oxide (ITO) anode (150 nm). The substrates were cleaned in consecutive ultrasonic baths of acetone, methanol, 2-propanol, and deionized water for 15 min each, and were then exposed to ultraviolet (UV) light under ozone atmosphere for 15 min. To form the hole injection layer (HIL), the substrates were spin-coated with poly(3,4-ethylenedioxythiophene): poly(styrene sulfonate) (PEDOT: PSS, Baytron P AI 4083) and baked at 150 °C for 10 min in air. The hole transport layer (HTL) was formed using blended poly-(3-hexylthiophene-2,5-diyl) (P3HT). After spin coating, HTL was baked at 80 °C for 30 min under vacuum conditions. Then, QDs were spin-coated to form a photoactive layer. Annealing was performed for 30 min at 80 °C under vacuum conditions. The electron transport layer (ETL) was formed by spin-coating zinc oxide (ZnO) NPs for 30 min at 90 °C under vacuum conditions. Finally, an aluminum (Al) cathode was deposited via thermal evaporation using a metal shadow mask. Subsequently, the active area of the fabricated device was defined to be 9 mm^2^. The I–V and J–V characteristics of SWIR photodetectors were determined using a parameter analyzer (B1500A, Agilent, Santa Clara, CA, USA).

## Results and discussion

**Fig. 1:**
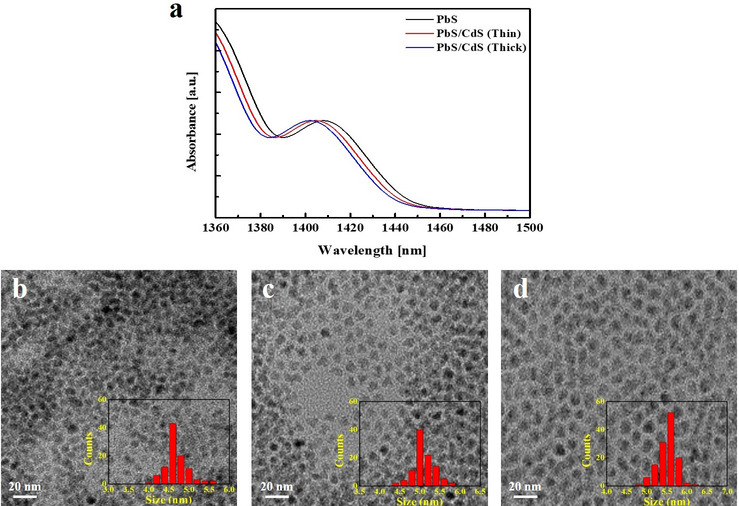
The characteristics of the synthesized QDs. **a** Absorption spectra of PbS, PbS/CdS (thin shell), and PbS/CdS (thick shell) QDs in toluene. TEM image and corresponding size histogram of the **b** PbS QDs, **c** PbS/CdS (thin shell), and **d** PbS/CdS (thick shell) QDs

PbS QDs were synthesized according to the previously reported literature [33, 38, 39], and then used to produce PbS/CdS core/shell QDs. The CdS shell was grown by the exchange of Pb^2+^ ions with Cd^2+^ ions at the surface of PbS QDs. According to Pietryga et al., regardless of the diameter of PbS QDs, the limiting shell thickness is not exceeded even with excess cadmium and longer reaction times [46]. Therefore, the efficiency of cation exchange is closely related to the effective diffusion of ions, which allows to grow thick shells at high temperatures. Figure 1a indicates that the growth of the CdS shell and the concomitant reduction of the PbS core result in a gradual blue-shift in the peak wavelength of the first exciton transition [33]. Although the total QD size distribution remains similar to that of PbS QDs regardless of the shell thickness, the core becomes non-uniform and more heterogeneous with an increase in shell thickness. Moreover, while excellent core homogeneity and uniform shell thickness are maintained when the shell is relatively thin, a thick shell can result in a core that is heterogeneous in both size and shape owing to the anisotropic nature of the cation exchange approach. The TEM images of synthesized QDs are presented in Fig. 1b–d. To grow thick shells, the core PbS QD can be stabilized against the Ostwald ripening process at high temperatures [36]. Two steps of the cation exchange reaction at different temperatures were needed to grow thick shells with a uniform size distribution. In the first step, a thin layer of CdS was deposited at 100 °C with a larger excess of cadmium than for the synthesis of thin shells. Then, cation exchange was allowed to proceed deeper into QDs by increasing the reaction temperature to 225 °C. This procedure strongly protects core PbS QDs and enables the high-temperature growth of thick-shell QDs with a uniform size distribution, as confirmed by TEM analysis. The size of PbS QDs is approximately 4.6 nm, as seen in TEM images. As the CdS surface shell is formed, an effective protective role can be expected; however, it is difficult to induce considerable shell growth by Cd because it has the characteristic of growing by cation exchange between Pb and Cd. TEM results show that there is no significant difference in particle size; however, there is a large change in optical properties [47]. Moffitt et al. have proposed an expression, which relates absorption wavelength ($\lambda_{a}$) to nanoparticle diameter ($D$) [48],

1 $${\text{D}} = 0.1 / \left( {0.1338 - 0.0002345 \lambda_{a} } \right)$$

The average sizes of PbS QDs, thin-shell PbS/CdS QDs, and thick-shell PbS/CdS QDs were estimated to be 4.6 $\pm$ 0.2, 5.0 $\pm$ 0.2, and 5.6 $\pm$ 0.1 nm, respectively.

**Fig. 2:**
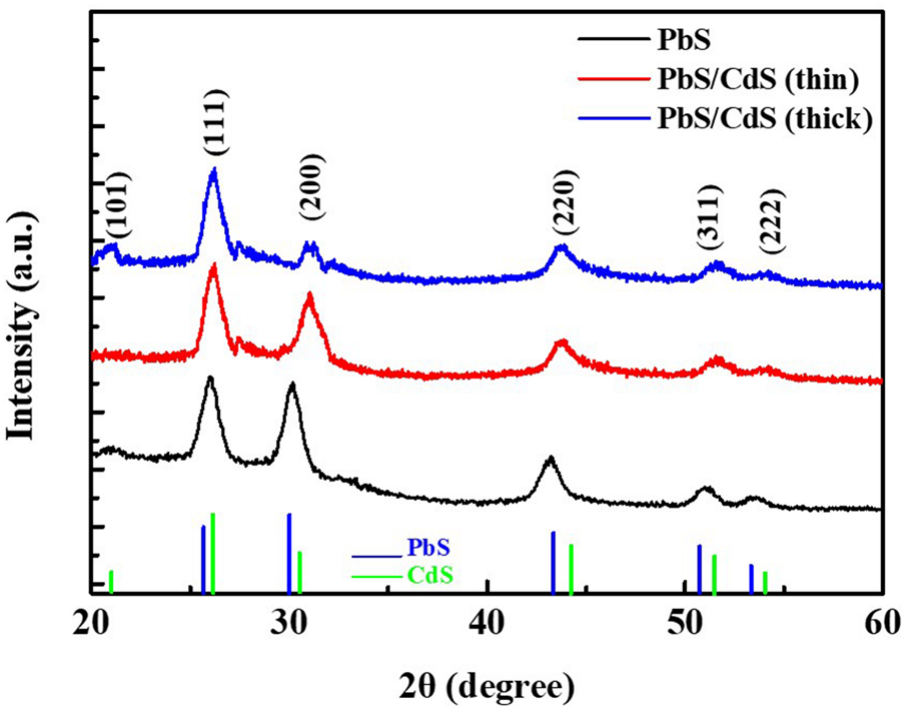
XRD patterns of PbS, PbS/CdS (thin shell), and PbS/CdS (thick shell) QDs. The JCPDS card files (05-0592, blue line) for PbS and (01 089 0440, green line) for CdS are shown below the spectra for identification

To confirm the formation of the CdS shell, X-ray diffraction (XRD) patterns were obtained. As shown in Fig. 2, the positions and relative peak heights of the patterns for PbS/CdS core/shell QDs are affected by the CdS shell on the PbS core. Bulk CdS has a higher peak intensity for (111) reflections compared to (200) reflections, whereas both the PbS QDs and PbS bulk phase have almost equal intensities for these reflections. The expected increased contribution of the shell layer to the overall composition, relative to that of remaining PbS, is confirmed by the increasing (111) peak intensity and decreasing (200) peak intensity with an increase in the CdS shell thickness. However, the decreased PbS pattern could not be detected owing to the weak signal from the small core. These results are consistent with those of Zhao et al. and Neo et al., who demonstrated that a much smaller bandgap is observed when an alloyed ${\text{Pb}}_{x} {\text{Cd}}_{1 - x}$ S phase shell is formed [39, 49]. In addition, the energy dispersive spectroscopy (EDS) analyses of PbS and PbS/CdS QDs shown in (Additional file 1: Fig. S2) demonstrate a decrease in the Pb ratio along with an increase in the Cd ratio as the thickness of the CdS shell increases. S^2−^ present in the CdS shell is supplied by PbS; however, there is no change in the absolute amount of S^2−^ ions between thin/thick shells. Because the component ratio of Cd^2+^ present in the cross section increases with an increase in Cd^2+^ ions injected from the solvent, the component ratio of Pb^2+^ and S^2−^ion decreases. Hence, the XRD and EDS results confirmed the formation of the CdS shell.

**Fig. 3:**
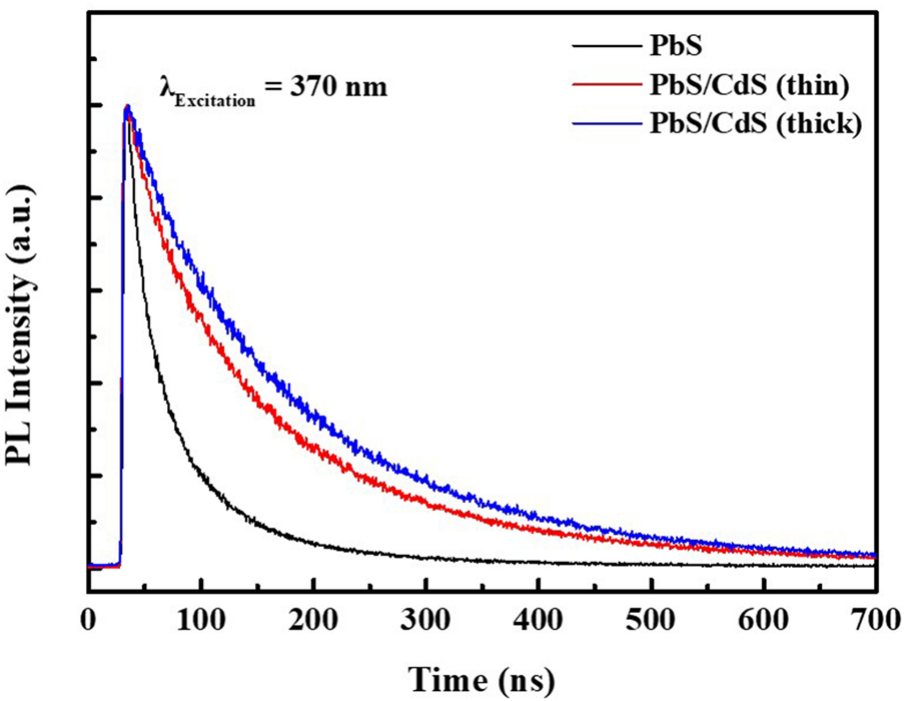
Time-resolved PL decay profiles of PbS, PbS/CdS (thin shell), and PbS/CdS (thick shell) QDs

To further understand the passivation provided by the shell material, we measured the time-resolved PL decay, as shown in Fig. 3. The results obtained for PbS QDs and thin- and thick-shell PbS/CdS QDs were fitted by a biexponential function with parameters for the amplitude (A) and decay time constant ($\tau$) [45]. As expected, the fastest PL decay was exhibited by PbS QDs owing to the high number of surface defects that feed the non-radiative recombination process [39, 45, 50–52]. The decreased rate of decay owing to the cation exchange process of core/shell PbS/CdS QDs was also confirmed, which demonstrates the effective passivation of PbS surface defects. The calculated average exciton lifetimes of PbS QDs and those of thin- and thick-shell PbS/CdS QDs were estimated to be 103.7, 220.3, and 261.1 ns, respectively.

**Fig. 4:**
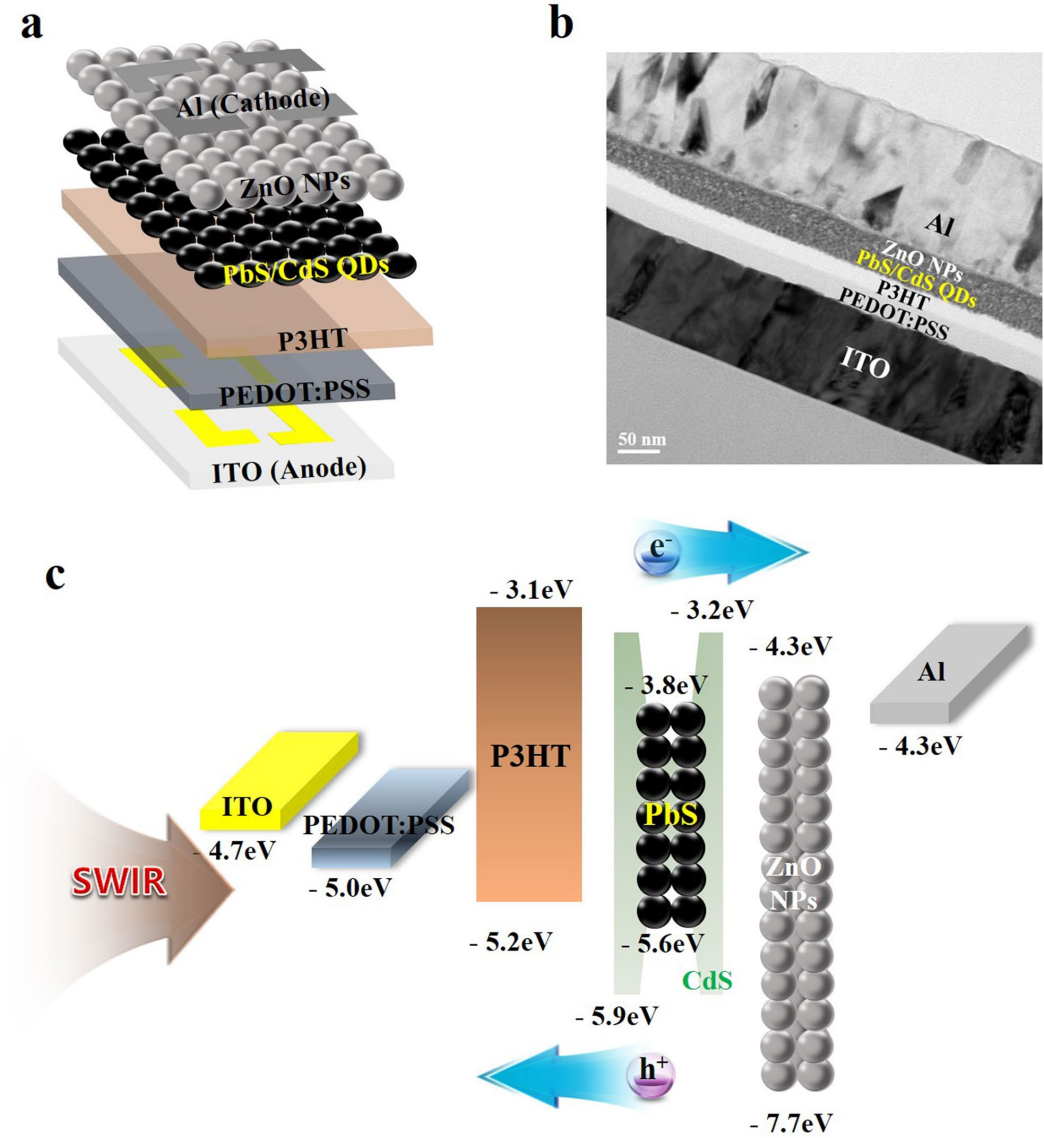
Device structure and TEM images of the all solution-processed SWIR photodetectors. **a** Schematic device structure, **b** cross-sectional (scale bar, 50 nm), and **c** energy band diagram of the all solution-processed SWIR photodetector

The device structure and corresponding energy level diagram are schematically shown in Figs. 4a, b. The device consists of patterned ITO as the anode, PEDOT:PSS as HIL, P3HT as HTL, QDs as the photoactive layer, ZnO NPs as ETL, and Al layer as the cathode. Except for the cathode, which was deposited using vacuum thermal evaporation, all other layers were sequentially deposited on the anode by a solution process. The fabrication of multilayered structure requires the use of orthogonal solvents to ensure the integrity of an underlying layer during the deposition of overlayers [43]. The vertical structure of photodetector provides both a high gain and a quick response owing to its small electrode spacing and a short carrier diffusion pathway.

**Fig. 5:**
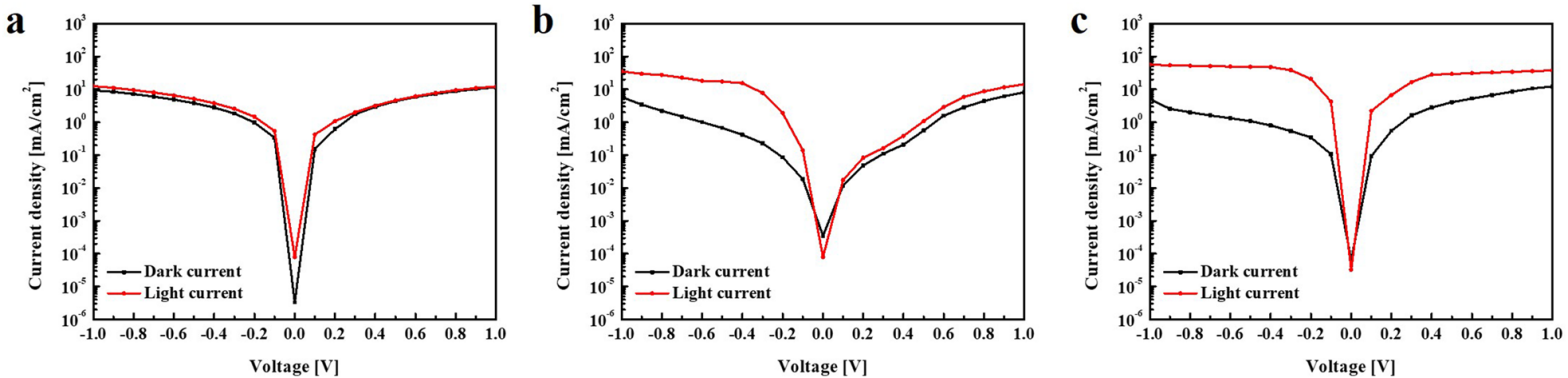
Current density–voltage ($J$-$V$) characteristics of fabricated SWIR photodetectors. **a** SWIR PD1, **b** SWIR PD2, and **c** SWIR PD3

The fabricated SWIR photodetector functions when incident light is absorbed in the active layer, and photoexcited electron–hole pairs (EHPs) are drawn to the electrodes by an external electric field. To analyze device performance, the J–V characteristics of three types of photodetectors in the range of –1 to 1 V, both in the dark and under IR illumination with a power density of 0.1 mW/cm^2^, are shown in Fig. 5. Three different devices were annotated as SWIR PD 1 (PbS QD-based SWIR photodetector), SWIR PD 2 (thin-shell PbS/CdS QD-based SWIR photodetector), and SWIR PD3 (thick-shell PbS/CdS QD-based SWIR photodetector). Under the reverse bias of − 1 V, the dark currents exhibited by SWIR PD1, SWIR PD2, and SWIR PD3 devices were respectively 9.32, 5.884, and 5.0526 mA/cm^2^, while the light currents were respectively 12.514, 35.3, and 56.856 mA/cm^2^. Hence. the J–V characteristics indicate a decrease in the relative dark currents of PbS/CdS QD-based SWIR photodetectors (SWIR PD2 and SWIR PD3) relative to that of the PbS QD-based SWIR photodetector (SWIR PD1). PbS/CdS QDs can induce surface trap states in the photoactive layer owing to the surface passivation afforded by the thickness of the CdS shell. However, the PbS/CdS QD-based SWIR photodetectors exhibited increased efficiency ratios of light current to dark current relative to that of PbS QD-based SWIR photodetector. As indicated in Table 1, the maximum-voltage on/off (light/dark) ratios of SWIR PD1, SWIR PD2, and SWIR PD3 devices were 1.34, 5.99, and 11.25, respectively. The core/shell PbS/CdS QDs are expected to increase the efficiency ratios of the corresponding SWIR photodetector IR illumination because CdS shells can decrease charge recombination in the photoactive layer owing to the charge trap formed by PbS/CdS QDs. Furthermore, dark current and light current are shown as a function of the applied bias in (Additional file 1: Fig. S3). The I–V curves show a diode-like behavior; with higher currents at high bias, the PbS/CdS QD-based SWIR photodetectors showed increased efficiency ratios of light current to dark current relative to that of a PbS QD-based SWIR photodetector.

**Table 1:** Performance of fabricated SWIR photodetector under IR light, 0.1 mW/cm2 at a bias of − 1 V

Device	J_dark_ (mA/cm^2^)	J_light_ (mA/cm^2^)	On/off ratio[J_light_/J_dark_] on bias of −1V	D^*^ [Jones]
SWIR PD 1	2.0	36,580	1.34	6.16 × 10^11^
SWIR PD 2	1.8	48,650	5.99	1.35 × 10^12^
SWIR PD 3	2.2	41,940	11.25	7.14 × 10^12^

The SWIR photodetector consisting of PbS/CdS QDs was evaluated regarding the effect of the CdS shell on the device performance, as evaluated using the detectivity (${\text{D}}$^*^) parameter in units of ${\text{J}}$ ones. This measurement allowed us to estimate the signal-to-noise ratio from the dark current, which has been previously shown to considerably contribute to noise [53, 54]. Detectivity can be expressed by the following equation:

2 $${\text{D}}^{ *} = \left( {\frac{{{\text{J}}_{\text{light}} - {\text{J}}_{\text{dark}} }}{{{\text{P}}_{\text{in}} }}} \right)/ \sqrt {2{\text{qJ}}_{\text{dark}} }$$

where ${\text{J}}_{\text{light}}$ and ${\text{J}}_{\text{dark}}$ are the light current under IR and the dark current, respectively; ${\text{P}}_{\text{in}}$ is the incident light intensity, and $q$ is the electron charge (1.6 $\times$ 10^−19^ C). The detectivities of SWIR PD1, SWIR PD2, and SWIR PD3 devices were 1.8 $\times$ 10^11^, 4.2 $\times$ 10^11^, and 4.0 $\times$ 10^12^${\text{J}}$ ones, respectively.

**Fig. 6:**
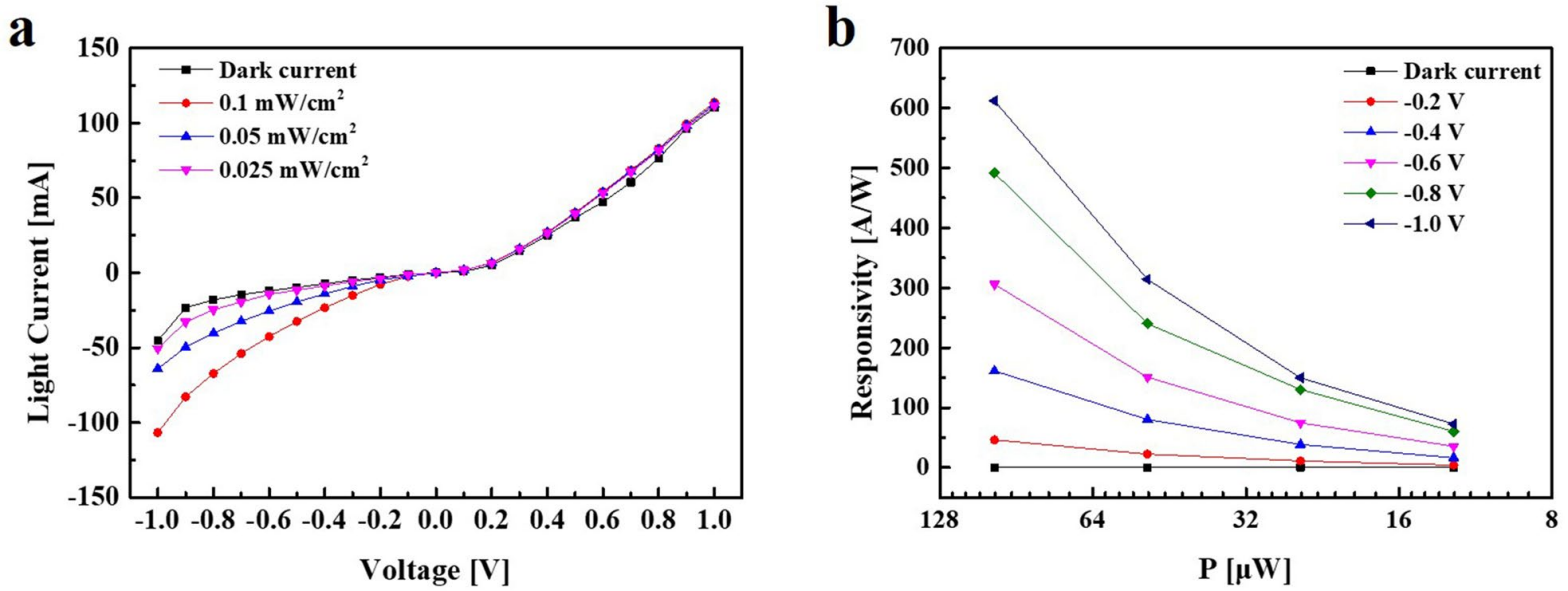
Dependence of photo-response on illumination intensity of SWIR PD3. **a** Current–voltage (I–V) characteristics at different illumination intensities using light (1400 nm wavelength), **b** the responsivities of the device as a function of incident powers at different bias voltages

To demonstrate the photoresponse capability of the device, we measured its response at different applied bias and incident light intensity. Under IR illumination the light current considerably increases, particularly at higher applied voltage, which demonstrates an asymmetrical and non-linear I–V behavior of the photodiode. The performance of the device under various incident powers (from 0.025 mW to 0.1 mW) is shown in Fig. 6a. The responsivity of the device, defined as $R = I_{ph} /P$, is calculated and shown in Fig. 6b. With a −1 V bias, the responsivity as high as 612 A/W is achieved under 1400 nm illumination with the incident power of 0.1 mW/cm^2^. The results indicated that SWIR PD1 showed the current difference of approximately 50 mA at −1 V. The power of light we used is much weaker than that of a previously reported PbS QD-based photodetector paper. It can be confirmed that the current scale is large in mA, which confirms that the performance of the PbS QD-based photodetector is excellent compared to other reported devices [3, 55].

**Fig. 7:**
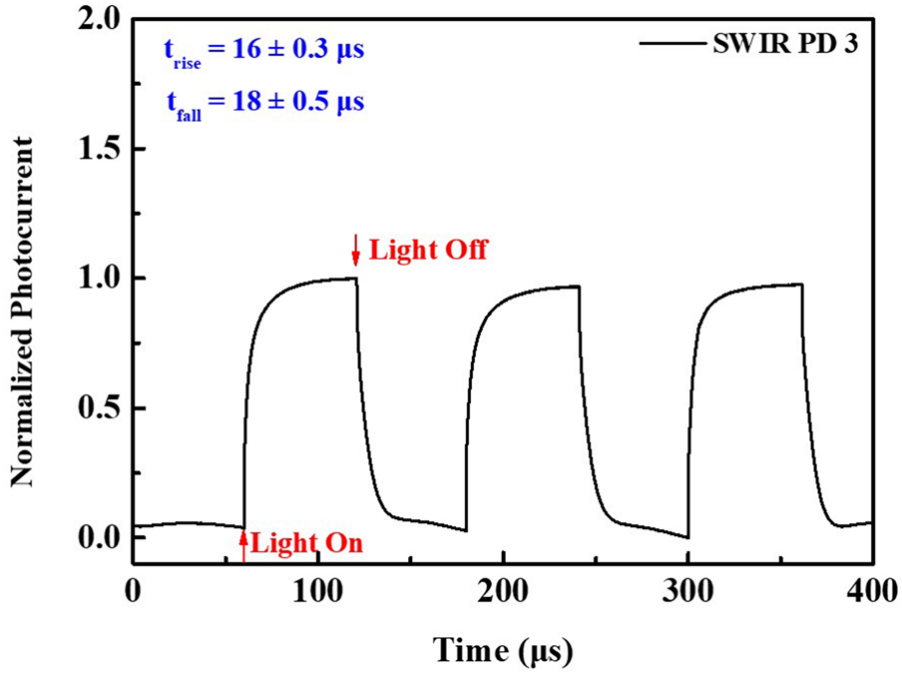
Transient photo-response waveform of PbS/CdS (thick shell) QD-based SWIR photodetectors (SWIR PD3) with bias of −1 V

The photoresponse speed provides information on carrier transport in the fabricated device. As shown in Fig. 7, the transient photocurrent of SWIR PD3 was measured under the bias of −1 V at the light intensity of 0.1 mW/cm^2^. The current rapidly increased under IR illumination and exponentially decreased without IR illumination. Furthermore, the device exhibited a steady response with repetitive IR illumination. The response time and fall times are defined as the times required to reach a 90% change in photocurrent with and without IR illumination, respectively [53, 54, 56, 57]. The results indicate that the response time of SWIR PD3 is 16 ± 0.3 µs, while the fall time is 18 ± 0.5 µs, which suggests that electron- or hole-blocking layers may also affect photocarrier collection. These data indicate that the fabricated device has a very fast response and fall time performance under IR illumination.

**Fig. 8:**
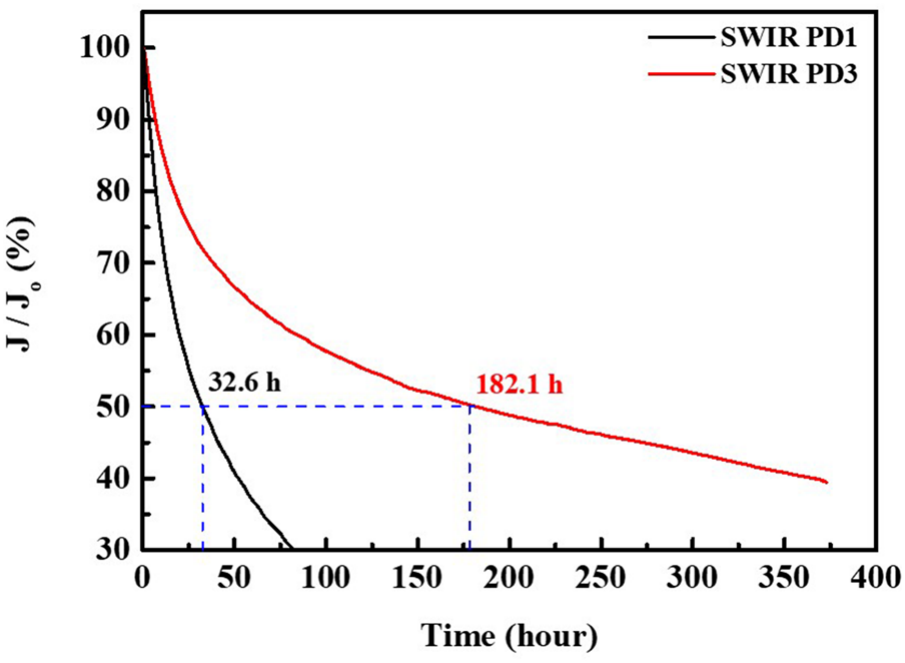
Lifetime characteristics of SWIR PD1 and SWIR PD3. Lifetime characteristics of SWIR PD1 (black line) and SWIR PD3 (red line) without encapsulation, under constant voltage operation − 1 V at room temperature

To validate the superior long-term stability of the device, the lifetimes of SWIR PD1 and SWIR PD3 devices were evaluated. The lifetime characteristics of unencapsulated SWIR photodetectors were assessed by operating devices at the constant voltage of –1 V under ambient conditions, as shown in Fig. 8. The lifetime T50 (measured in hours) is the time required for the current density to decrease to 50% of its initial value. At 0.1 mW/cm^2^ of continuous operation, the current density of the SWIR PD1 device rapidly deteriorated from initial 12.514 mA/cm^2^ to reach T50 after 32.6 h of continuous operation, whereas that of the SWIR PD3 device slowly decayed from initial 56.856 mA/cm^2^ to reach T50 after 182.1 h. These results are very promising given that the device would be operating at room temperature (25 °C) for the majority of applications (medical and bio imaging, detection of objects, and human–machine interfaces) [58]. In addition, these results indicate that the SWIR PD3 device is more stable under operating conditions and that its lifetime is almost 5.5 times higher compared to that of the SWIR PD1 device. The excellent stability of the PbS/CdS (thick shell) QD-based SWIR photodetector (SWIR PD3) is attributed to the efficient QD passivation afforded by the thick CdS shell, which serves as a physical barrier to the penetration of oxygen.

## Conclusions

In conclusion, we designed, fabricated, and optimized a solution process for a SWIR photodetector that was based on type-I core/shell PbS/CdS QDs. The TEM, XRD, and EDS results confirm the successful production of the CdS shell, which leads to the considerable enhancement in detectivity, photoresponse time, and long-term stability of the device. PbS/CdS QDs with a thick shell were synthesized via a two-step cation exchange approach. A thicker shell offers the PbS core better protection from the environment along with enhanced photostability. The resulting solution-processable thick-shell PbS/CdS QD-based SWIR photodetector exhibited excellent performance, with a high on/off (light/dark) ratio of 11.25, high detectivity of 4.0 $\times$ 10^12^ Jones, fast response (110 ms) and fall (133 ms) times, and operative lifetime of 182.1 h. The considerable improvements in electrical performance and stability of the device were attributed to the self-passivation characteristics of the thick CdS shell, which serves as a physical barrier to the penetration of oxygen and moisture. Hence, thick-shell PbS/CdS QDs are potentially applicable to a wide range of optoelectronic applications owing to their SWIR absorption, long-lifetime, capacity for photo-induced charge transfer, and fast photoresponse.

## Supplementary information

**Additional file 1: Fig. S1.** The characteristics of synthesized ZnO NPs. **a** UV–Vis absorption spectrum and **b** TEM image. **Fig. S2.** EDS spectra and elemental compositions (insets) of QDs. **a** PbS QDs, **b** thin-shell PbS/CdS QDs, and **c** thick-shell PbS/CdS QDs. **Fig. S3.** Current-voltage (I–V) characteristics of fabricated SWIR photodetectors. a PbS QD-based SWIR photodetector, b PbS/CdS thin shell QD-based SWIR photodetector, and c PbS/CdS thick shell QD-based SWIR photodetector.

## Data Availability

The datasets used and/or analyzed during the current study are available from the corresponding author on reasonable request.

## Abbreviations

QD(s): Quantum dot(s)
LED(s): Light-emitting diode(s)
IR: Infrared
PV: Photovoltaic
SWIR: Short-wave infrared
PbS: Lead sulfide
PL: Photoluminescence
PL QY(s): Photoluminescence quantum yield(s)
J–V: Current density–voltage
PbCl_2_: Lead chloride
S: Sulfur
OLA: Oleylamine
CdO: Cadmium oxide
OA: Oleic acid
1-ODE: 1-octadecene
TOP: Trioctylphosphine
Zn(acet)_2_·2H_2_O: Zinc acetate dehydrate
KOH: Potassium hydroxide
NP(s): Nanoparticle(s)
TEM: Transmission electron microscopy
ITO: Indium tin oxide
UV: Ultraviolet
HIL: Hole injection layer
PEDOT:PSS: Poly(3,4-ethylenedioxythiophene): poly(styrene sulfonate)
HTL: Hole transport layer
P3HT: Poly-(3-hexylthiophene-2,5-diyl)
ETL: Electron transport layer
ZnO: Zinc oxide
Al: Aluminum
I–V: Current-voltage
XRD: X-ray diffraction
EDS: Energy dispersive spectroscopy
EHP(s): Electron–hole pair(s)
D^*^: Detectivity
