# Supplementary material for: Air-stable and ultrasensitive solution-cast SWIR photodetectors utilizing modified core/shell colloidal quantum dots
Source: Nano Converg. 2020 Aug 17;7:28. doi: 10.1186/s40580-020-00238-3 (PMC7429620; doi:10.1186/s40580-020-00238-3)
Supplement: Supplementary file 1 — Additional file 1: Fig. S1. The characteristics of synthesized ZnO NPs. a UV–Vis absorption spectrum and b TEM image. Fig. S2. EDS spectra and elemental compositions (insets) of QDs. a PbS QDs, b thin-shell PbS/CdS QDs, and c thick-shell PbS/CdS QDs. Fig. S3. Current-voltage (I–V) characteristics of fabricated SWIR photodetectors. a PbS QD-based SWIR photodetector, b PbS/CdS thin shell QD-based SWIR photodetector, and c PbS/CdS thick shell QD-based SWIR photodetector. [file 40580_2020_238_MOESM1_ESM.docx]

Supplementary Information

Air-stable and ultrasensitive solution-cast SWIR photodetectors utilizing modified core/shell colloidal quantum dots

Jin-Beom Kwon^1^, Sae-Wan Kim^1^, Byoung-Ho Kang^2^, Se-Hyuk Yeom^2^, Wang-Hoon Lee^2^, Dae-Hyuk Kwon^3^, Jae-Sung Lee^2^* and Shin-Won Kang^1^*

*^1^School of Electronics Engineering, College of IT Engineering, Kyungpook National University, 1370 Sankyuk-dong, Daegu, 702-701, Republic of Korea*

*^2^Advanced Semiconductor Research Center, Gumi Electronics & Information Technology Research Institute (GERI), Gumi 39253, Republic of Korea*

*^3^Department of Electronic Engineering, Kyungil University, Hayang-up, 712-702, Gyeongsang buk-do, Republic of Korea*

*Author to whom correspondence should be addressed. E-mail: jslee1245@geri.re.kr, swkang@knu.ac.kr

**1. Synthesis and purification of zinc oxide (ZnO) nanoparticles**

A modification of the sol-gel method was used for the synthesis of ZnO nanoparticles (NPs) in alcohol solution. The solutions were prepared using 2.46 g of Zn(acet)_2_·2H_2_O and 0.96 g of KOH dispersed in 110 and 50 mL of methanol, respectively. The Zn(acet)_2_·2H_2_O solution was placed in a 200 mL flask and heated to 60 °C and the KOH solution was added by droplets (1 mL/s). The mixture was stirred at 60 °C for 60 min and then allowed to cool. To obtain uniform ZnO NPs, we implemented the necessary aging process by adding 2-propanol and hexane overnight. The ZnO NPs were then precipitated via centrifugation at 3,000 rpm and re-dispersed in ethanol (30 mg/mL). To determine the characteristics of the ZnO NPs, we performed UV-visible spectrum analysis and transmission electron microscopy (TEM), as shown in Fig. S1.


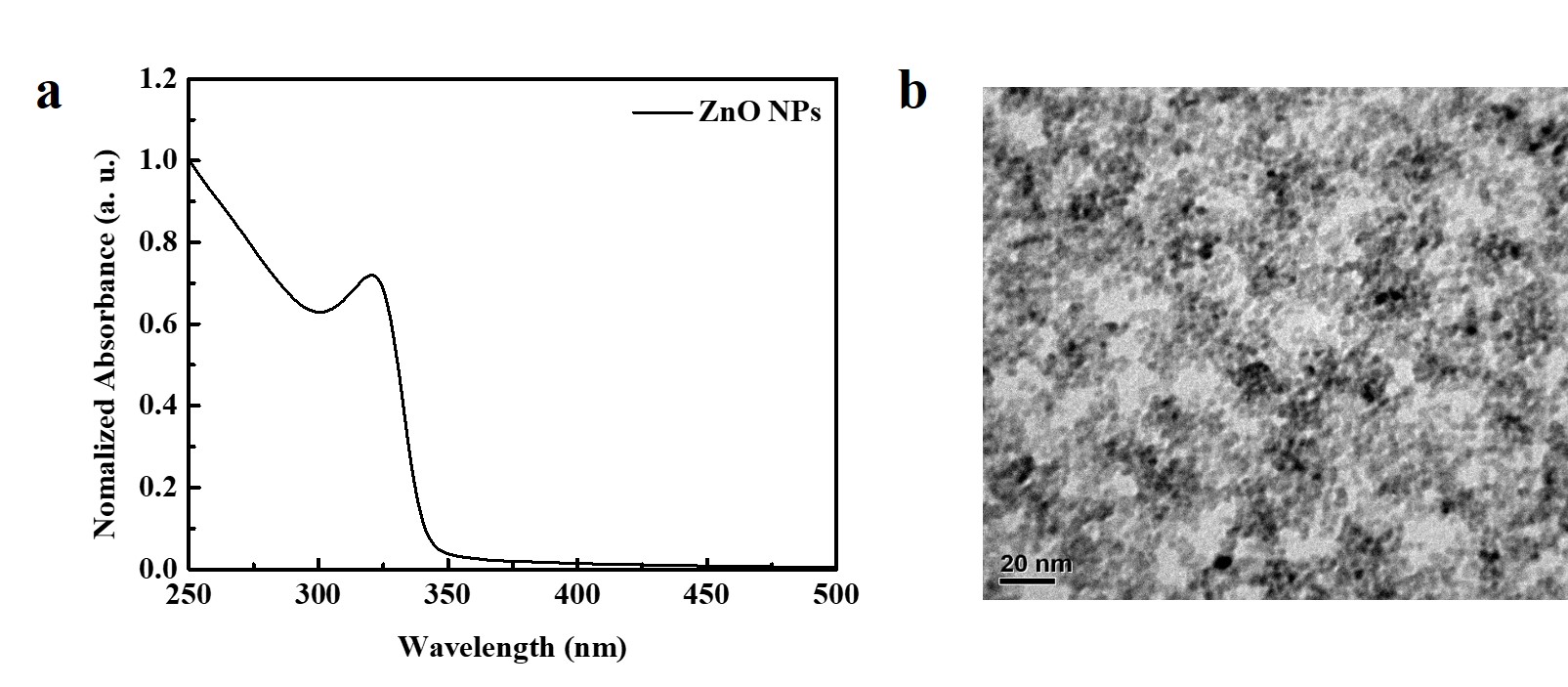


**Fig. S1** The characteristics of synthesized ZnO NPs. **a** UV-Vis absorption spectrum and **b** TEM image

**2. Component analysis of PbS QDs, the thin-shell PbS/CdS QDs, and the thick-shell PbS/CdS QDs**

The energy dispersive spectroscopy (EDS) analyses of the PbS and PbS/CdS QD presented in Fig. S2 demonstrate a decreased pb ratio along with an increased Cd ratio as the thickness of the CdS shell increased. PbS supplies S^2-^ present in the CdS shell but there is no change in the absolute amount of S^2-^ ions between the thin/thick shells. Because the component ratio of Cd^2+^ in the cross- section increases with the increase of Cd^2+^ ions injected from the solvent, the component ratio of Pb^2+^ and S^2-^ion decreases.


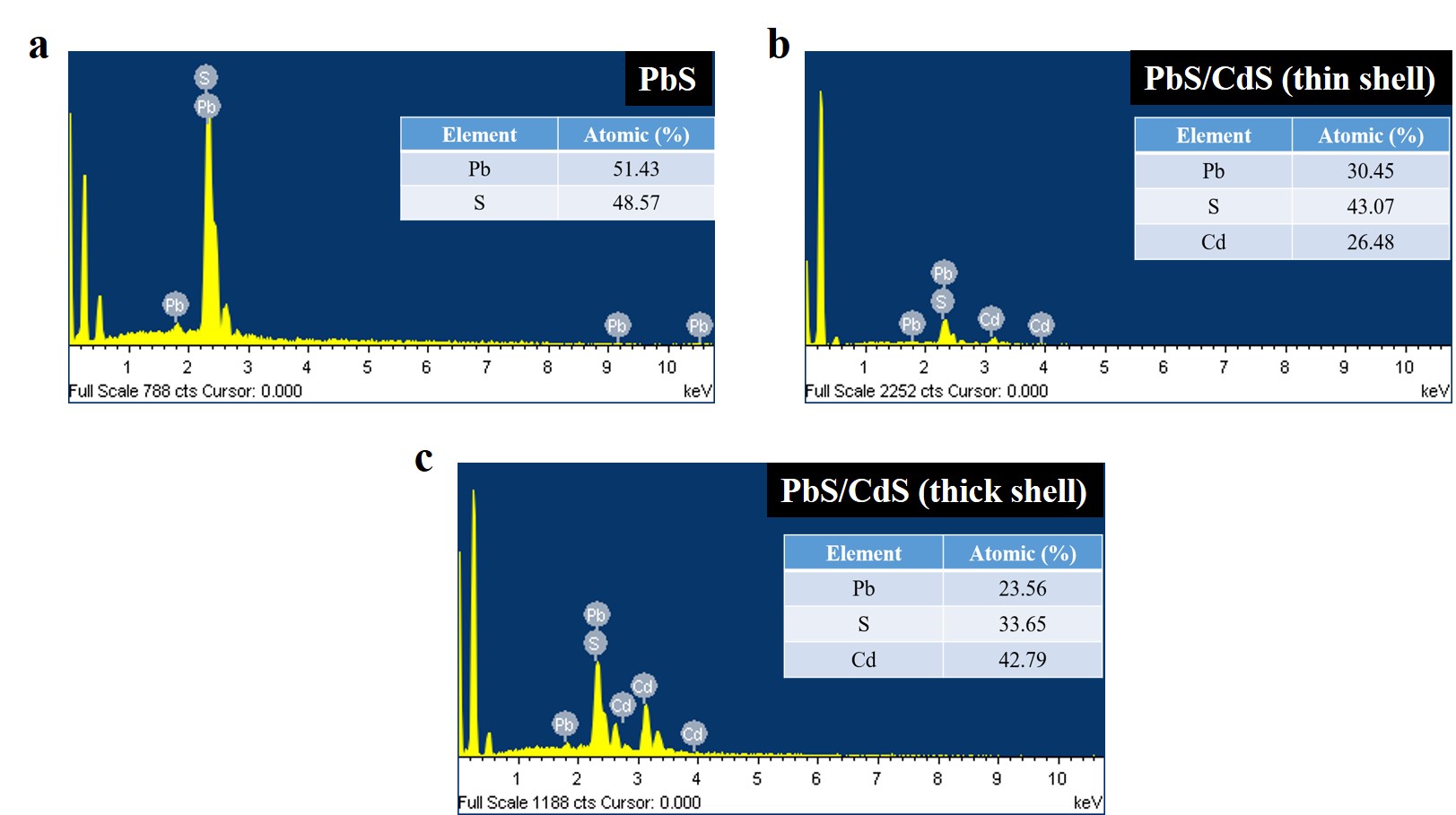


**Fig. S2** EDS spectra and elemental compositions (insets) of QDs. **a** PbS QDs, **b** thin-shell PbS/CdS QDs, and **c** thick-shell PbS/CdS QDs.

**3. Current-voltage (I-V) characteristics of PbS QD-based SWIR photodetector, PbS/CdS thin shell QD-based SWIR photodetector, and PbS/CdS thick shell QD-based SWIR photodetector**

The dark current and light current are shown as a function of the applied bias in Fig. S3. The data were taken using a parameter analyzer (B1500A, Agilent, Santa Clara, CA, USA). The I-V curves show diode-like behavior, with higher currents at high bias. The PbS/CdS QD-based SWIR photodetectors displayed increased efficiency ratios of light current to dark current, relative to that of the PbS QD-based SWIR photodetector.


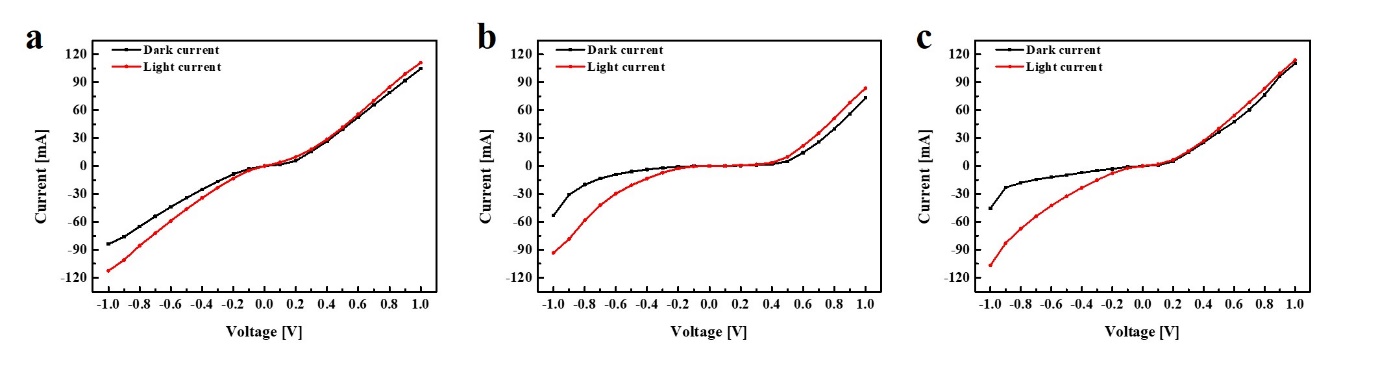


**Fig. S3** Current-voltage ($I$-$V$) characteristics of fabricated SWIR photodetectors. a PbS QD-based SWIR photodetector, b PbS/CdS thin shell QD-based SWIR photodetector, and c PbS/CdS thick shell QD-based SWIR photodetector.
